# Shwachman–Diamond syndrome due to biallelic *EFL1* variants with complex and fatal clinical course in early infancy

**DOI:** 10.1111/bjh.19793

**Published:** 2024-10-08

**Authors:** Holger Cario, Alexis Bertrand, Shengjiang Tan, Bernd Auber, Miriam Erlacher, Eva‐Maria Mair, Sandra von Hardenberg, Dirk Lebrecht, Patrick Revy, Alan J. Warren

**Affiliations:** ^1^ Department of Pediatrics and Adolescent Medicine University Medical Center Ulm Ulm Germany; ^2^ Center for Rare Hematopoietic Disorders and Immunodeficiencies (ZSHI), Rare Disease Center University Medical Center Ulm Ulm Germany; ^3^ Laboratory of Genome Dynamics in the Immune System INSERM UMR 1163, Imagine Institute Paris France; ^4^ Université Paris Cité, Imagine Institute Paris France; ^5^ Université Paris‐Saclay Paris France; ^6^ Cambridge Institute for Medical Research, University of Cambridge, Cambridge Biomedical Campus Cambridge UK; ^7^ Wellcome Trust‐Medical Research Council Stem Cell Institute, Jeffrey Cheah Biomedical Centre, Puddicombe Way, Cambridge Biomedical Campus Cambridge UK; ^8^ Department of Haematology Jeffrey Cheah Biomedical Centre, Cambridge Biomedical Campus, University of Cambridge School of Clinical Medicine Cambridge UK; ^9^ Department of Human Genetics Hannover Medical School Hannover Germany; ^10^ Center for Pediatrics and Adolescent Medicine, Pediatric Hematology and Oncology University Medical Center Freiburg Freiburg Germany; ^11^ Division of Neonatology and Pediatric Intensive Care Medicine, Department of Pediatrics and Adolescent Medicine University Medical Center Ulm Ulm Germany

**Keywords:** bone marrow failure, EFL1, infancy, Shwachman–Diamond syndrome

## Abstract

Shwachman–Diamond syndrome represents a clinically and genetically heterogeneous disorder. We report on an infant with a very severe, fatal clinical course caused by biallelic *EFL1* variants: c.89A>G, p.(His30Arg), and c.2599A>G, p.(Asn867Asp). Functional analysis of patient‐derived B‐lymphoblastoid and SV40‐transformed fibroblast cell lines suggests that the compound heterozygous *EFL1* variants impaired mature ribosome formation leading to compromised protein synthesis, ultimately resulting in a severe form of Shwachman–Diamond syndrome.

## BACKGROUND

Shwachman–(Bodian)–Diamond syndrome (SBDS, hereafter denoted SDS) is a very rare disorder with an estimated incidence of around 0.5–1.5/10^5^ live births.^1^ When the eponymous authors described the clinical picture of SDS,^2^, ^3^ it appeared to be a clearly defined disease with uniform clinical presentation comprising severe neutropenia and failure to thrive due to pancreatic insufficiency.

Fittingly, the first gene associated with this autosomal recessive inherited disorder was given the name of *SBDS*, apparently also on the assumption that the clearly defined phenotype would also be based on a largely homogenous genotype.

Although *SBDS* mutations are responsible for about 90% of cases, we now know that genetic alterations leading to SDS or SDS‐like phenotypes may also be caused by pathogenic variants in *DNAJC21*, *SRP54* and *EFL1*.^4^, ^5^, ^6^, ^7^, ^8^

Most importantly, we know that the phenotypic spectrum is much broader than the original definition of SDS suggested and includes isolated neutropenia, developmental delay, skeletal changes and increased propensity to develop haematopoietic neoplasms. Phenotypic differences related to the functional consequences of the specific genetic variant have been described.^1^, ^9^, ^10^ However, predicting clinical severity is difficult, particularly in cases associated with compound heterozygous variants. This is further complicated by the fact that at the somatic level, there may be spontaneous genetic alterations which modify the expected phenotype,^11^ as recently exemplified by a *loss of heterozygosity* in favour of the ‘milder’ of the two variants in a subset of blood cells from a patient with EFL1‐associated SDS.^12^

Next‐generation sequencing analyses often provide apparently reliable causal explanations at an early stage of the diagnostic work‐up of ambiguous clinical presentations. However, variants of uncertain significance are often detected that have not yet been linked to the disease and for which experimental support for their functional relevance is lacking.

Against this background, a clear differential diagnosis and the careful comparison of the phenotype with cases reported elsewhere are of great importance.

For these reasons, we report on the case of an infant with fatal SDS with complex clinical symptoms and underlying compound heterozygous *EFL1* mutations.

## CASE

A female child of non‐consanguineous parents was born preterm after 35 + 3/7 gestational weeks in an external hospital. She presented small for gestational age with a birth weight 1400 g (<3. centile), a height of 40 cm (<3. centile), a head circumference of 30.5 cm (6. centile), prenatally diagnosed oligohydramnion with secondary lung hypoplasia, atrial septal defect (ASD) II, high‐arch palate, slightly shortened limbs and general hypotonia. Pulmonary insufficiency necessitated surfactant administration and assisted ventilation with various modalities. Early‐onset severe pulmonary arterial hypertension was treated with nitric oxide, sildenafil and bosentan. After extubating at the age of 3 weeks, further ventilatory support with continuous positive airway pressure (CPAP) followed by high‐flow therapy was provided. The latter was continued in combination with sildenafil treatment after discharge at the age of 10 weeks.

At birth, the child presented with pancytopenia of varying severity (initial haemoglobin 110 g/L, neutrophils 0.3 × 10^9^/L, platelets 50 × 10^9^/L) necessitating repeated transfusions of erythrocytes and thrombocytes until the time of discharge.

Numerous examinations to exclude metabolic disorders and pancreatic faecal elastase were performed without significant pathological results. Abdominal ultrasound did not show specific changes apart from splenomegaly. There were no specific skeletal abnormalities on thoracic X‐ray. Echocardiography showed features of ASD II and pulmonary hypertension.

At the age of 3 months, the girl was admitted to our institution due to severe pancytopenia and increasing oxygen requirement, resulting in severe pulmonary insufficiency. Due to additional recurrent cardiac decompensation, treatment with catecholamines and diuretics was given but did not lead to sustained improvement. Structural lung disease in addition to secondary hypoplasia was suspected (biopsy not done). The need for assisted ventilation, later via tracheostomy, persisted. At the age of 9 months, the patient was discharged to an institution specialized in palliative care of such patients including assisted ventilation and transfusions. There, the child died at the age of 18 months due to cardiopulmonary failure during an acute viral infection.

Gastrointestinal and feeding problems early on necessitated parenteral nutrition via central venous catheter, which subsequently changed to enteral nutrition via percutaneous endoscopic gastrostomy. Responding to later results consistent with exocrine pancreatic insufficiency, supplementation with pancreatic enzymes and fat‐soluble vitamins was initiated. Later in the clinical course, the patient in addition developed endocrine pancreatic insufficiency with impaired glucose tolerance.

Severe pancytopenia of varying severity persisted. The patient received frequent transfusions of erythrocytes and thrombocytes. Treatment with G‐CSF was initiated because of very severe neutropenia associated with systemic and cutaneous infections. There was no evidence for haemolysis or an infectious or immunological cause of the thrombocytopenia and neutropenia. Reticulocyte count, soluble transferrin receptor, mean platelet volume and the percentage of immature platelets were low, consistent with bone marrow failure (BMF). Bone marrow examination revealed hypocellularity and hyposegmented neutrophils (pseudo‐Pelger anomaly), and no increased vacuolization, blasts or haemophagocytosis. Bone marrow cytogenetics were normal.

Considering the initial normal pancreatic elastase measurements, further investigations first concentrated on various causes of neonatal BMF syndromes other than SDS. These included telomere length analysis, functional testing for Fanconi anaemia, followed by focused genetic testing for *SAMD9*, *SAMD9L*, *GATA2* and *RUNX1* mutations and then extended to a selection of genes involved in infantile myelodysplastic syndromes and BMF. Mitochondrial genetic alterations were also excluded.

## FURTHER INVESTIGATIONS AND RESULTS

Severe exocrine pancreatic insufficiency was diagnosed at the age of 5 months, with significantly reduced faecal pancreatic elastase values (<15 μg/g, normal >200). *SBDS*‐sequencing analysis revealed a wild‐type sequence. SDS diagnostics were extended to other genes which participate in the final steps of ribosome maturation and proper translation, including *DNAJC21*, *SRP54* and *EFL1*.^9^ Two heterozygous variants were detected in the *EFL1* gene: NM_024580.6: c.89A>G, p.(His30Arg) and c.2599A>G, p.(Asn867Asp). Segregation analysis of the parents confirmed the compound heterozygous state. The affected amino acids, His30 and Asn867 (NP_078856.4), are evolutionarily highly or moderately conserved respectively. EFL1 residue His30 lies within the G1 motif, also known as P‐loop, that binds to the α‐ and β‐phosphates of GTP (Figure S2), while residue Asn867 maps to domain IV (Figure S2), which is important for functional interaction with the SBDS protein.^13^, ^14^ The frequency of the *EFL1*:c.89A>G p.(His30Arg) variant was 1.05e^−5^ in the normal population according to the gnomAD database (V4.1.0) while the *EFL1*:c.2599A>G p.(Asn867Asp) variant was absent in this database (Table 1). Furthermore, these variants were not listed in the ClinVar and LOVD databases at the time of the study.

**TABLE 1:** Genotype and phenotype data of published cases with EFL1‐related SDS and the new case.

Patient	Variant sequence	Variant protein	Sex	Phenotype	Outcome^a^	Ref
1 (Fam I)	c.2645T>A homozygous	Met882Lys	m	Term birth. Neonatal hypotonia, subglottic stenosis, laryngeal cleft, laryngomalacia, umbilical hernia and poor feeding. At 7 months of age, short stature, developmental delay and metaphyseal dysplasia (ribs and femurs). At age 2, failure to thrive, pancreatic insufficiency with intermittent diarrhoea and constipation. Later, frequent infections, severe myopia and global developmental delay. Haematology: chronic neutropenia, transient anaemia and thrombocytopenia. BM normal	Alive, 6 years	[7]
2 (Fam I)	c.2645T>A homozygous	Met882Lys	f	Short stature, severe myopia, mild speech delay, short stature, metaphyseal dysplasia of lower extremities and pancreatic insufficiency (but normal weight). Haematology: intermittent neutropenia. BM normal	Alive, 4 years	[7]
3 (Fam II)	c.3284G>A homozygous	Arg1095Gln	f	Low birth weight, hypotonia, microcephaly, low set ears, high‐arch palate, limb and finger shortening. Metaphyseal dysplasia (ribs and limbs). Severe failure to thrive. Pancreatic insufficiency with severe diarrhoea and steatorrhoea. Haematology: progressively profound neutropenia, progressive anaemia and fluctuating thrombocytopenia. BM with trilineage hypoplasia	Died, 14 months	[7]
4 (Fam II)	c.3284G>A homozygous	Arg1095Gln	m	Same phenotype as patient 3 incl. haematology	Died, 15 months	[7]
5 (Fam II)	c.3284G>A homozygous	Arg1095Gln	m	Same phenotype as patient 3 but responding to enzyme replacement. Developmental delay. Haematology: profound neutropenia and anaemia, fluctuating thrombocytopenia. BM with trilineage hypoplasia	Alive, 15 months	[7]
6	c.3284G>A homozygous	Arg1095Gln	f	Same phenotype as patient 3 including haematology	Died, 7 mo.	[7]
7	c.379A>G homozygous	Thr127Ala	f	Low birth weight. Mild neonatal thrombocytopenia. Failure to thrive. Hepatopathy at age 2–4. Metaphyseal dysplasia limbs and vertebrae; scoliosis with subsequent restrictive lung disease. Normal psychomotor development. No gastrointestinal symptoms, only very mild exocrine pancreatic insufficiency, diagnosis at age 14 based on WES data. Haematology: mild and intermittent thrombocytopenia, no anaemia and no neutropenia except one episode related to viral illness. BM with trilineage hypoplasia	Alive, 14 years	[16]
8	c.[2260C>T]; [1514T>C]	Arg754^a^ Phe505Ser	m	Term birth, SGA. Later: unsteady gait, spontaneous fracture at age 5. Genu valgum. Metaphyseal chondrodysplasia was diagnosed at age 13. Mild intellectual impairment. At age 27, chronic diarrhoea and pancreatic insufficiency. Liver cirrhosis. Haematology: at age 17, mild thrombocytopenia, progressive neutropenia and mild anaemia. At age 31, BM with hypocellularity and hyposegmented neutrophils	Alive, 31 years	[8]
9	c.2908C>T homozygous	Arg970His	f	Term birth, SGA. At 13 months of age, failure to thrive, anorexia, elevated liver enzymes, reduced vitamin levels and iron deficiency anaemia. At age 1.5, ataxia and nystagmus. At age 3.8, neutropenia, thrombocytopenia and exocrine pancreatic insufficiency. Very severe myopia. Normal intellectual development. Haematology: iron deficiency anaemia, mild thrombocytopenia and later neutropenia. BM with hyposegmented neutrophils	Alive, 11 years	[8]
10	c.2647T>G/? defective EFL1 expression	Cys883Gly/?	f	Term birth, SGA, pectus carinatum, short limbs, severe neonatal anaemia and thrombocytopenia and increased liver enzymes. Pancreatic insufficiency with chronic diarrhoea at 3 months of age. Anorexia and growth retardation. Chondrodysplasia of vertebrae, limbs and ribs. Impaired cognitive development. Haematology: Transfusion‐dependent anaemia until age 2, transfusion independent thereafter. BM with erythroblastopenia and hyposegmented neutrophils	Alive, 7 years	[8]
11	c.[2478dupT]; [3205A>G]	Gly827Trpfs13 Thr1069Ala	m	Preterm birth, SGA. Pancytopenia at 2 months of age. Metaphyseal dysplasia. Pancreatic insufficiency with lipomatosis. Haematology: Pancytopenia. BM with hypocellularity, reduced megakaryopoiesis and increased storage iron	Alive, 3 years	[12]
12	c.[89A>G]; [3205A>G]	His30Arg Thr1069Ala	f	Preterm birth, SGA. Pancreatic lipomatosis, metaphyseal dysplasia, osteopenia, short stature	Alive, 9 years	[12]
13	c.[89A>G]; [3205A>G]	His30Arg Thr1069Ala	m	Low birth weight. At age 2, exocrine and endocrine pancreatic insufficiency, metaphyseal chondrodysplasia and ichthyosis. Later osteoporosis and pancreatic lipomatosis. Haematology: anaemia, thrombocytopenia and intermittent neutropenia since age 2	Alive, 25 years	[12]
14	c.[89A>G]; [2599A>G]	His30Arg Asn867Asp	f	Preterm birth, SGA. Prenatal oligohydramnion. ASD II, high‐arch palate and slight limb shortening. Pulmonary hypertension, pulmonary insufficiency, lifelong assisted ventilation and tracheostomy performed. Suspected structural lung disease. Severe neonatal and persisting pancytopenia. Exocrine pancreatic insufficiency was diagnosed at 5 months of age, later also endocrine dysfunction. Gastrostomy for enteral feeding. Further information is in the main text. Haematology: Severe neonatal and persisting pancytopenia. GCSF for very severe neutropenia and recurrent infections. BM with hypocellularity and hyposegmented neutrophils	Died, 18 months	This report

Abbreviations: BM, bone marrow; SGA, small for gestational age; WES, whole exome sequencing.

^a^ For alive patients, age at the time of the report is given.

An in silico analysis using MobiDetails^15^ suggested a pathogenic impact for the variant p.(His30Arg) and a more heterogeneous picture for the variant p.(Asn867Asp; Figure S1).

**FIGURE 1:**
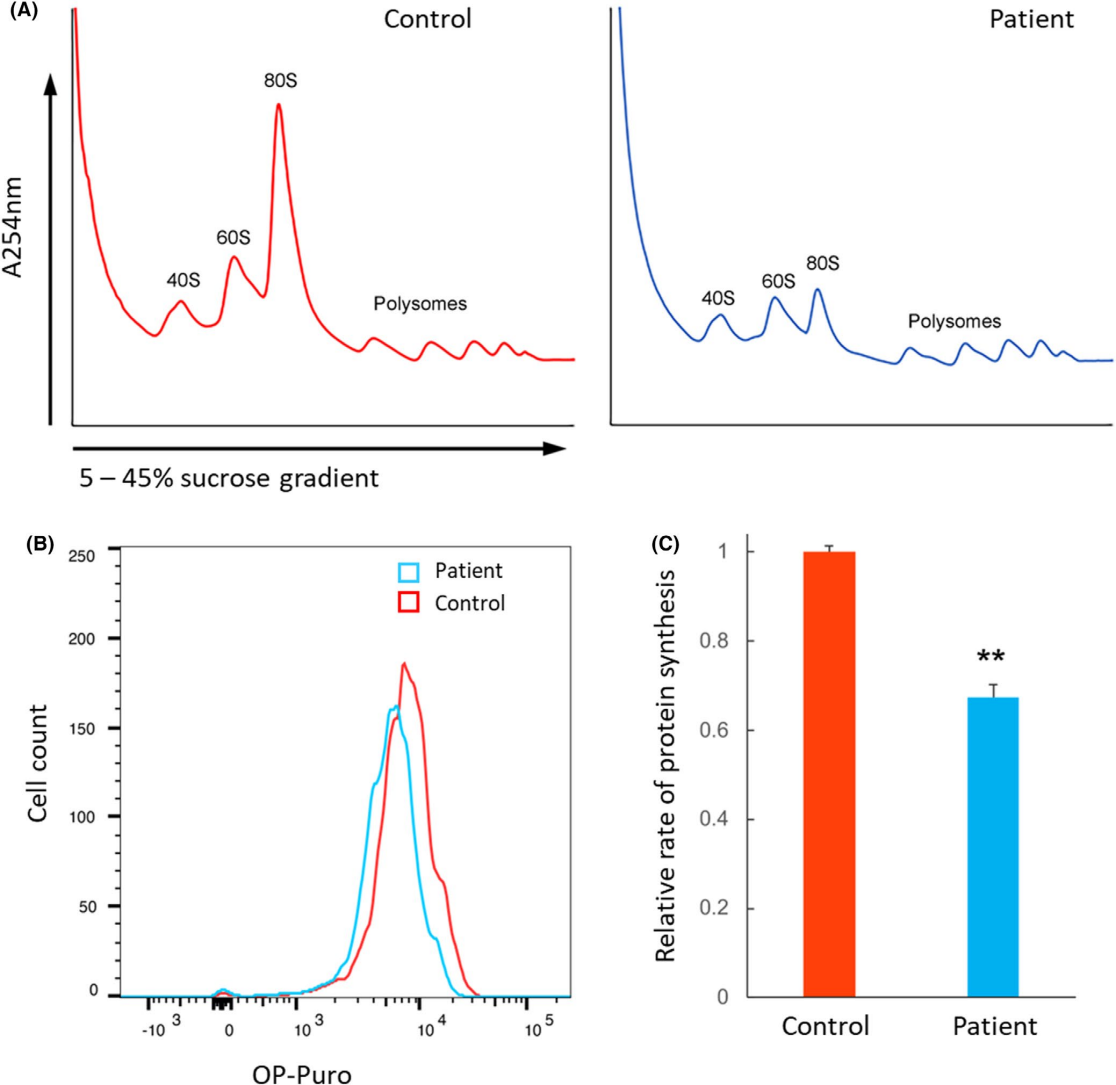
Patient‐derived cells carrying compound heterozygous *EFL1* p.(His30Arg), p.(Asn867Asp) variants have attenuated ribosome assembly and protein synthesis. (A), Sucrose gradient sedimentation of fibroblast cell extracts from wild‐type control and patient. (B, C) OP‐Puro incorporation into patient‐derived fibroblasts quantified by flow cytometry indicates reduced global protein synthesis in EFL1‐deficiency patient cells relative to wild‐type control cells (**PT test = 0.0006, three replicate; Supplementary Methods,^8^).

To assess the effect of the *EFL1* variants, we established a patient‐derived EBV (Epstein Barr virus)‐infected B‐lymphoblastoid cell line (B‐LCL) and an SV40‐transformed fibroblast cell line (SV40‐FB). The similar signal obtained from the EFL1 immunoblot of extracts from control and patient‐derived B‐LCL and SV40‐FB indicated that the variants did not affect the expression and/or stability of the protein (Figure S3). Furthermore, SBDS, NMD3, eIF6 and DNAJC21, which are critical factors involved in the final steps of the ribosomal maturation process, were similarly expressed in cells from the patient and healthy donors (Figure S3). However, consistent with impaired ribosomal assembly, the polysome profile obtained from the sucrose gradient of the patient's cells showed a strong reduction in mature 80S ribosomes (Figure 1A). Consistent with this result, the patient's SV40‐FB showed a significant reduction in the rate of global protein synthesis compared to controls as assessed by incorporation of O‐propargyl‐puromycin (OP‐Puro; Figure 1B,C). Collectively, these functional data suggest that the compound heterozygous *EFL1* variants caused impaired mature ribosome formation leading to compromised protein synthesis, ultimately resulting in a severe form of SDS.

## DISCUSSION

Our results strongly argue for a causal link between the patient's severe SDS disease and the biallelic *EFL1* variants. Although, unfortunately, this result had no impact on the patient's fatal outcome, it allowed clinical and genetic counselling for the parents.

The EFL1:p.(His30Arg) variant has since been described in two further patients in a study which also provided data on the functional effects of this variant on mature ribosome production (Table 1).^12^ In both patients, an EFL1:p.(Thr1069Ala) variant was present on the second allele. One patient showed uniparental disomy for this functionally milder variant in the haematopoietic system. As a result, this patient, 9 years old at the time of the report, had several non‐haematological clinical features, but normal blood cell counts. In contrast, the second patient, 25 years old at the time of the report, presented with varying pancytopenia in addition to pancreatic insufficiency and metaphyseal chondrodysplasia.^12^

In comparison with the other previously published paediatric cases with EFL1‐related SDS (Table 1), the clinical severity of our patient is reminiscent of that of the four patients with homozygous EFL1:p.(Arg1095Gln) in the first description of *EFL1* variants underlying SDS (Table 1).^7^ These patients had severe failure to thrive, skeletal abnormalities, pancreatic insufficiency with severe diarrhoea and marked blood count changes. At the time of publication, three of these four patients had already died at the age of less than 2 years, while one 15‐month‐old patient was still alive.

A patient with EFL1:p.(Cys883Gly), presumably in compound heterozygosity with a variant in non‐coding regions of the *EFL1* gene, had a similar early onset of symptoms as our patient.^8^ Neonatal presentation with growth retardation, short limbs, severe anaemia, thrombocytopenia and liver disease was reported (Table 1). Pancreatic insufficiency was diagnosed at the age of 3 months. Transfusion‐dependent anaemia persisted until 2 years of age. At the time of the report, the patient was 7 years old and presented without any significant blood count changes but with general physical and neurological developmental delay.

The fatal course in our patient in early infancy contrasts not only with the less severe clinical phenotypes previously described in the two SDS patients carrying the same heterozygous EFL1:p.(His30Arg) variant in compound heterozygosity with a second milder variant^12^ but also with disease severity in the other reported cases with bi‐allelic *EFL1* mutations except those with homozygous EFL1:p.(Arg1095Gln) (Table 1).^7^

It is unclear to what extent the patient's suspected structural lung disease, which is presumably responsible for the ultimately fatal course of the disease, was related to the EFL1 functional impairment. While the other clinical symptoms have been repeatedly described in other SDS patients, such lung involvement has not been reported previously. Suitable models, for example, modified induced pluripotent stem cells (iPSCs), are necessary to address these questions and to investigate the role and tissue‐specific effects of pathogenic variants in *EFL1* and the other SDS‐related genes.

## AUTHOR CONTRIBUTIONS

HC, PR and AJW designed the case presentation. HC wrote the manuscript. PR and AJW both contributed essential parts to the manuscript. AB and PR established lymphoblastoid and fibroblast cell lines and performed western blot experiments. ST and AJW performed cell culture experiments, ribosome subunit analysis and measurement of protein synthesis. AJW added alpha fold modelling of mutated residues. ME and DL were essentially involved in the haematological and genetic work‐up on BMF syndrome and MDS exclusion. SvB and BA performed and evaluated SDS genetics and whole‐exome analysis. EMM and HC were responsible for the clinical care of the patient and for diagnostic procedures and collected and evaluated clinical and lab data. All authors read, commented, corrected and finally, accepted the manuscript.

## PATIENT CONSENT STATEMENT

N/A: ‘photographs or any part of the body that could identify the patient’ were not used.

## Supporting information


Figure S1.

Figure S2.

Figure S3.

Table S1.
