# Supplementary figures and images for: Shwachman–Diamond syndrome due to biallelic EFL1 variants with complex and fatal clinical course in early infancy
Source: Br J Haematol. 2024 Oct 8;205(6):2363–9. doi: 10.1111/bjh.19793 (PMC11637716; doi:10.1111/bjh.19793)

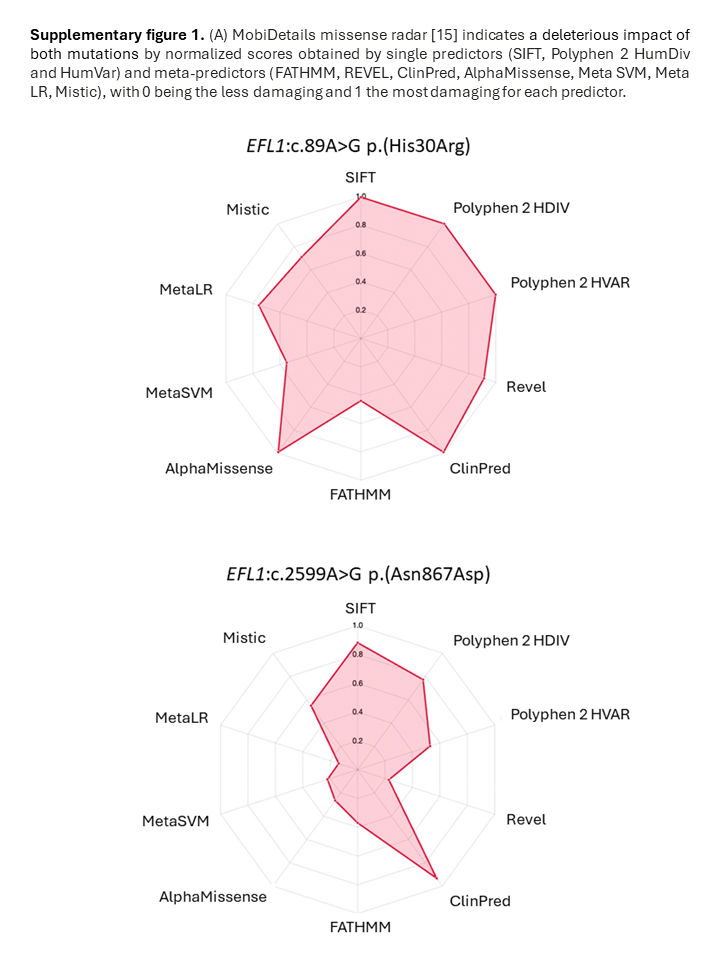

Supplement: Supplementary file 1 — Figure S1. [file BJH-205-2363-s003.tif]

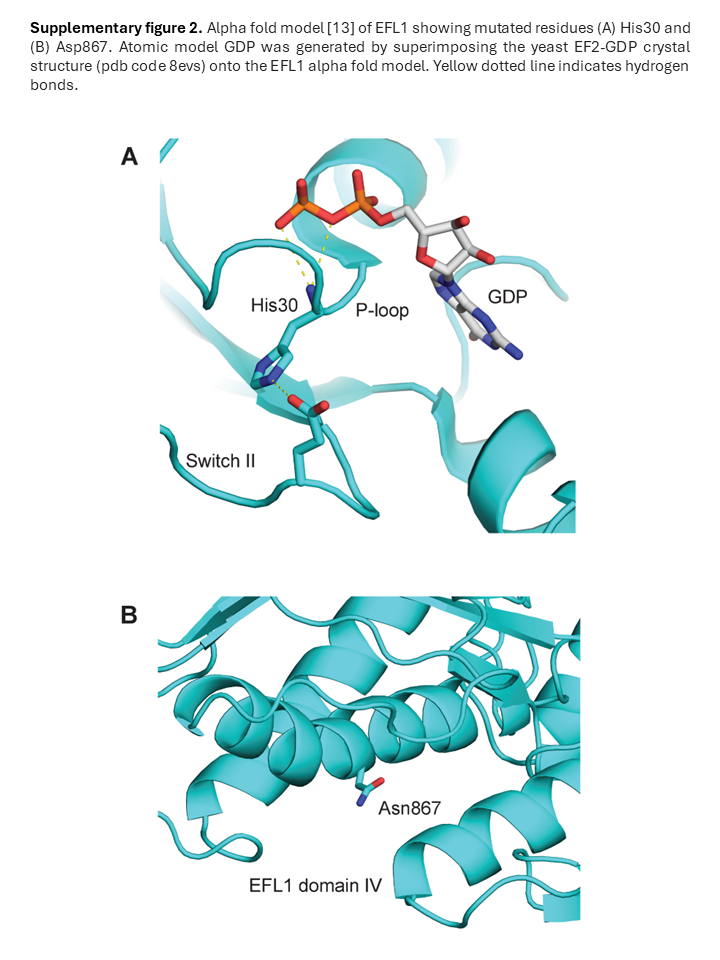

Supplement: Supplementary file 2 — Figure S2. [file BJH-205-2363-s002.tif]

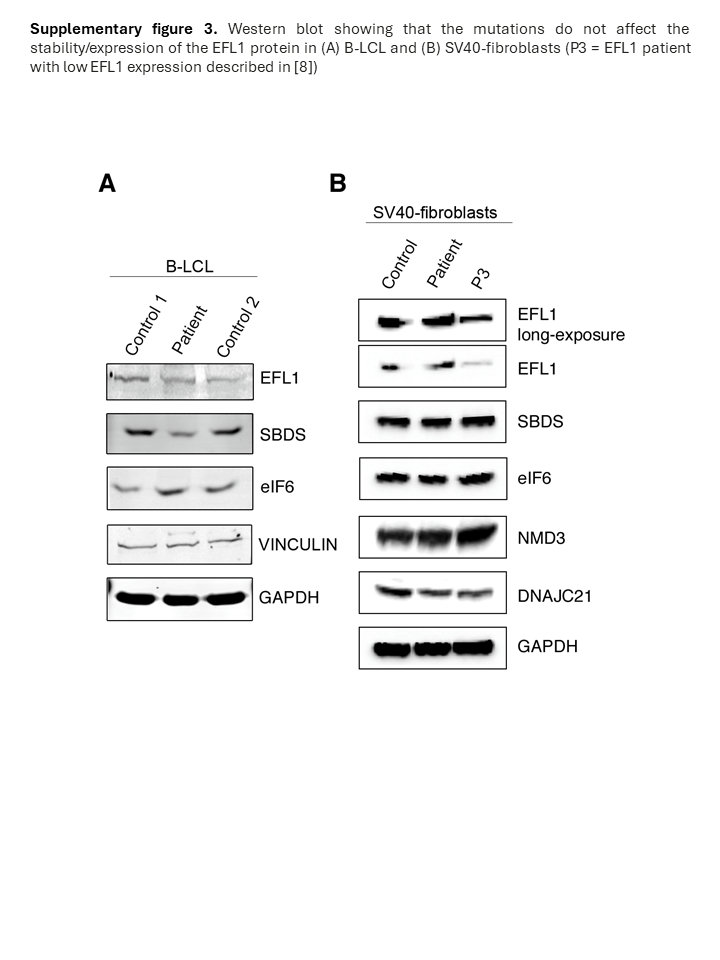

Supplement: Supplementary file 3 — Figure S3. [file BJH-205-2363-s001.tif]
