# Supplementary material for: Shwachman–Diamond syndrome due to biallelic EFL1 variants with complex and fatal clinical course in early infancy
Source: Br J Haematol. 2024 Oct 8;205(6):2363–9. doi: 10.1111/bjh.19793 (PMC11637716; doi:10.1111/bjh.19793)
Supplement: Supplementary file 4 — Table S1. [file BJH-205-2363-s004.docx]

**Supplementary Methods**

**Cell culture.** Fibroblast cell lines and B-lymphoblastoid cell lines (B-LCL) were cultured at 37 °C with 5 % CO_2_ in DMEM and RPMI Medium 1640 (Gibco™ GlutaMAX™), respectively, supplemented with 10 % fetal bovine serum and 1 % Penicillin-Streptomycin (Sigma).

**Immunoblotting.** Cell pellets were lyzed in RIPA buffer (20 mM HEPES at pH 7.4, 20 mM β-glycerophosphate, 10 mM NaF, 0.5 mM EDTA, 0.5 mM EGTA, 0.2 M NaCl, 1 % [v/v] Nonidet P-40, 0.5 % [w/v] sodium deoxycholate, 0.1 % [w/v] SDS) with complete EDTA-free protease inhibitors (Roche), and incubation on ice for 10 min with occasional vortexing. Cell lysates were cleared in a microfuge and normalized for protein concentration using a BCA protein assay kit (Pierce). Protein samples were separated on SDS-PAGE gels and transferred to PVDF membranes. Antibodies are listed in Supplementary Table 1 below. Protein bands were visualized either by chemiluminescence (SuperSignal West Pico Chemiluminescent Substrate, ThermoFisher) or fluorescence (Li-COR, Odyssey CLx). Specific protein signal was measured using Fiji (Image J)1 and ImageStudio (Li-COR).

**Sucrose gradient sedimentation.** Ribosomal subunits were separated by sucrose density gradients as previously described [1-5]. Briefly, fibroblasts (60 % - 80 % confluence) were treated with with cycloheximide at final concentration of 100 μg /mL for 15 min at 37 °C before harvesting. Cells were then lyzed in lysis buffer (20 mM HEPES pH 7.4, 50 mM KCl, 5 mM MgCl_2_, 0.5 % (v/v) IGEPAL^®^ CA-630 (Sigma, #I8896), 0.5 % (w/v) Sodium deoxycholate, 100 µg/mL cycloheximide (Sigma, #C7698) with complete EDTA-free protease inhibitors (Roche) and 0.5 U/mL RNase inhibitor (Invitrogen) and incubated for 15 min on ice. Lysates were cleared in a microcentrifuge. Equal amounts (typically 2 A_254_ U) were applied to a 10-40 % (w/v) sucrose gradient in 14 mL of buffer B (20 mM HEPES at pH 7.4, 50 mM KCl, 5 mM MgCl_2_) and centrifuged (Beckman SW40 rotor) at 284, 600 *g* for 2 hr at 4 °C). Samples were loaded on a Brandel gradient fractionator and polysome profiles detected using an ÄKTAprime plus system (GE Healthcare).

**Measurement of protein synthesis.** Protein synthesis was measured as described [3-5]. Briefly, OP-Puro (Invitrogen; final concentration 50 μM) was added to the culture medium (Dulbecco's Modified Eagle Medium (DMEM, Gibco™ GlutaMAX™), 10 % fetal bovine serum (Sigma) and 1 % Penicillin-Streptomycin (Pen-Strep, Sigma)) for 60 min. Cells were collected and washed twice in ice-cold Ca^2+^- and Mg^2+^-free phosphate buffered saline (PBS) (Invitrogen) with 100 μg/mL cycloheximide. Cells were then fixed and permeabilized using the Cytofix/Cytoperm Fixation Permeabilization Kit (BD Biosciences). Azide-alkyne cycloaddition was performed using the Click-iT Cell Reaction Buffer Kit (Invitrogen) with azide conjugated to Alexa Fluor 488 at 5 μM final concentration. Following the 30 min reaction, cells were washed twice in PBS supplemented with 2% fetal bovine serum, resuspended in PBS and analyzed by flow cytometry (Becton Dickinson LSR Fortessa analyzer). Flow cytometry data analysis was performed using FlowJo v10.1 (FlowJo, Ashland, OR). ‘Relative rates of protein synthesis’ were calculated by normalizing OP-Puro signal to control cells after subtracting background fluorescence (cells without OP-Puro).

**Statistics**

Student's t test (two-tailed, type 2) was used to determine significant differences. P< 0.05 was considered significant.

**Supplementary Table 1. Antibodies.**

| **Name** | **Source** | **Application** |
| --- | --- | --- |
| anti-DNAJC21 | Aviva, #ARP62801 | IB |
| anti-EFL1 | Abgent, #AP10373B | IB |
| anti-eIF6 | GenTex, #GTX117971 | IB |
| anti-GAPDH | Sigma, #G9545 | IB |
| anti-NMD3 | Proteintech, #16060-1-AP | IB |
| anti-SBDS | Finch et al 2011  Santa-Cruz SC-271350 | IB |
| anti-mouse IgG HRP | Sigma, #A5278 | IB |
| anti-rabbit IgG HRP | Cell Signaling, #7074 | IB |
| anti-Vinculin | Santa-Cruz SC-271350 | IB |

**Supplementary methods references**

1. Finch, A.J. et al. Uncoupling of GTP hydrolysis from eIF6 release on the ribosome causes Shwachman-Diamond syndrome. Genes Dev 2011; 25: 917-29. doi:10.1101/gad.623011
2. Wong, C.C. et al. Defective ribosome assembly in Shwachman-Diamond syndrome. Blood 2011; 118: 4305-12. doi:10.1182/blood-2011-06-353938
3. Tan, S. et al. EFL1 mutations impair eIF6 release to cause Shwachman-Diamond syndrome. Blood 2019; 134: 277-290. doi:10.1182/blood.2018893404.
4. Tan, S. et al Somatic genetic rescue of a germline ribosome assembly defect. Nat Commun. 2021; 12: 5044. doi:10.1038/s41467-021-24999-5.
5. Jaako, P. eIF6 rebinding dynamically couples ribosome maturation and translation. Nat Commun. 2022; 13: 1562. doi: 10.1038/s41467-022-29214-7.
